# Association Between Cardiovascular Risk and Subclinical Atherosclerosis in Korean Female Patients with Systemic Lupus Erythematosus

**DOI:** 10.3390/jcm14207162

**Published:** 2025-10-11

**Authors:** Ju-Yang Jung, Jaemi Kim, Ji-Hyun Park, Bumhee Park, Ji-Won Kim, Hyoun-Ah Kim, Chang-Hee Suh

**Affiliations:** 1 Department of Rheumatology, Ajou University School of Medicine, Suwon 16499, Republic of Korea; serinne20@hanmail.net (J.-Y.J.); funny1126@hotmail.com (J.K.); jwk722@naver.com (J.-W.K.); nakhada@naver.com (H.-A.K.); 2 Office of Biostatistics, Medical Research Collaborating Center, Ajou Research Institute for Innovative Medicine, Ajou University School of Medicine, Suwon 16499, Republic of Korea; jhn1105@gmail.com (J.-H.P.); bhpark@ajou.ac.kr (B.P.); 3 Department of Biomedical Informatics, Ajou University School of Medicine, Suwon 16499, Republic of Korea

**Keywords:** systemic lupus erythematosus, carotid artery ultrasonography, cardiovascular risk score, cholesterol

## Abstract

**Background**: Cardiovascular disease (CVD) is a major complication of systemic lupus erythematosus (SLE). This study compared several CV risk scores in Korean female patients with SLE and searched for an association with subclinical atherosclerosis and lipid metabolism. **Methods**: Female SLE patients and healthy controls (HCs) underwent carotid ultrasonography and pulse wave velocity (PWV), and serum efflux cholesterol capacity was measured. The Framingham risk scores (FRSs), American College of Cardiology/American Heart Association (ACC/AHA) scores, and Korean Risk Prediction Model (KRPM) scores were calculated. **Results**: While carotid intima-media thickness (IMT) and the prevalence of carotid plaque did not differ between 67 SLE patients and 37 HCs, carotid plaque scores were higher in SLE patients compared with HCs. While the FRS and the ACC/AHA CV risk scores did not differ, the KRPM scores were higher in SLE patients. The carotid IMT, plaque score, and PWV were correlated with the FRS, ACC/AHA CV risk, and KRPM score in SLE patients. SLE patients with carotid plaque had higher FRS, ACC/AHA CV risk, and KRPM scores than those without carotid plaque. In addition, the serum cholesterol efflux capacity did not differ between SLE patients with and without carotid plaque but was correlated with carotid IMT. **Conclusions**: The scores obtained from the CV risk-prediction models were correlated with subclinical atherosclerosis in SLE. A cardiovascular risk assessment tool developed specifically for Koreans is suitable for evaluating the CV risk in Korean SLE patients.

## 1. Introduction

Systemic lupus erythematosus (SLE) is a chronic autoimmune disease characterized by the production of autoantibodies and immune complex deposition in various organs, including the skin, joints, and kidneys [1]. Recurrent inflammatory responses and the long-term use of immunosuppressive therapy often lead to organ damage and comorbidities. Among these, early-onset cardiovascular disease (CVD) is a leading cause of morbidity and mortality in SLE, with patients exhibiting a substantially higher prevalence of cardiovascular complications than the general population [2,3]. In the Framingham cohort, the prevalence of myocardial infarction was reported to be five-fold higher in middle-aged women with SLE than in healthy controls [4]. Similarly, large cohort studies have demonstrated a 3.7-fold increased incidence of stroke and more than a four-fold higher risk of cardiovascular mortality in patients with SLE [5,6]. Recent nationwide data further confirmed that Korean patients with SLE carry nearly double the risk of CVD compared with the general population, with an adjusted incidence rate ratio of 1.99, exceeding even that of patients with diabetes [7].

The increased risk of atherosclerosis in SLE is multifactorial. Chronic systemic inflammation, long-term glucocorticoid exposure, and microvascular dysfunction contribute to vascular injury, while conventional risk factors such as hypertension, dyslipidemia, and smoking further exacerbate CVD risk [3,8,9,10]. Because SLE often develops at a relatively young age, accurate identification of patients at high cardiovascular risk is clinically important for prevention and management.

Several cardiovascular risk prediction tools, including the Framingham risk factor score (FRS), the 2013 American College of Cardiology/American Heart Association (ACC/AHA) pooled cohort equations, and the System for Cardiac Operative Risk Evaluation (SCORE), are widely used in the general population [11,12,13]. These models incorporate traditional risk factors, such as age, sex, blood pressure, lipid profile, smoking status, and diabetes, to guide preventive interventions [13]. However, their applicability in SLE remains uncertain. The Korean Risk Prediction Model (KRPM), developed using the Korean Genome and Epidemiology Study, has shown a superior predictive accuracy for atherosclerotic CVD in Koreans compared with the FRS and the ACC/AHA equations [14,15].

To enhance the prediction of future CV events, especially in individuals without clinically apparent disease, several imaging-based techniques have been introduced. These include carotid intima-media thickness (IMT), carotid plaque assessment, and pulse wave velocity (PWV), which evaluates arterial stiffness and flow dynamics [16,17,18]. The vascular changes detected by these tools, which precede clinical cardiovascular events, are referred to as subclinical atherosclerosis.

High-density lipoprotein (HDL) contributes to cardiovascular protection by promoting reverse cholesterol transport from macrophage [19]. Not only the quantity of HDL but also its functionality of HDL plays a critical role in atherosclerosis. One key functional parameter of HDL is its ability to promote cholesterol efflux from lipid-laden macrophages—a process termed cholesterol efflux capacity—which can be measured using patient serum [20]. Cholesterol efflux capacity (CEC) had been found to be inversely associated with CV events in the general population [21,22]. CEC had been dysregulated in patients with SLE, but it was attenuated by type I IFN receptor inhibition with anifrolumab [23,24,25]. In addition, HDL atheroscleroprotective functions presented by cholesterol efflux and antioxidant capacities of HDL were improved after anti-B cell activating factor monoclonal antibody in patients with SLE [26].

Given these considerations, it is crucial to evaluate the performance of existing CV risk prediction tools in SLE, their association with imaging-based markers of subclinical atherosclerosis, and the potential role of HDL functionality in vascular health. In this study, we compared conventional and population-specific CV risk models in patients with SLE, correlated them with vascular imaging findings, and examined the relationship between CEC and markers of subclinical atherosclerosis.

## 2. Materials and Methods

### 2.1. Study Design

Sixty-seven female patients with SLE, who met the Systemic Lupus International Collaborating Clinics/American College of Rheumatology (SLICC/ACR) classification criteria, and thirty-seven age-matched healthy controls (HCs) were recruited for the study [27]. Patients with SLE and HCs aged 40 years or older but younger than 65 years were enrolled. No patients had malignancy, infection, or other autoimmune diseases, and all were under treatment in the rheumatology clinic of Ajou University.

Laboratory data—including blood cell counts, complement levels, and anti-double-stranded DNA (dsDNA) antibodies—and data on clinical manifestations, such as oral ulcer, malar rash, alopecia, arthritis, and renal disease, were collected. Disease activity and disease-related damage were assessed using the SLE Disease Activity Index (SLEDAI) and the SLICC/ACR Damage index, and medication information was collected from medical records. In addition, data on traditional CV risk factors and demographic features were collected. Informed consent form and study protocols were reviewed and approved by the Institutional Review Board of Ajou University Hospital (AJIRB-SUR-2018-466), in accordance with the Declaration of Helsinki [28].

### 2.2. Ascertainment of FRS, ACC/AHA Risk Score, and KRPM

The FRS, 2013 ACC/AHA CV risk score, and KRPM scores of the participants were calculated using age; total cholesterol, and high-density lipoprotein (HDL), low-density lipoprotein (LDL) levels; systolic BP; treatment for hypertension; diabetes mellitus status; and smoking status [11,12,13,14,15]. We used the white version of the 2013 ACC/AHA CV risk score.

### 2.3. Assessment of Subclinical Atherosclerosis

Carotid artery intima-media thickness (IMT) and plaque were assessed by high-resolution B-mode ultrasonography (Accuvix XG, Samsung Medison, Seoul, Republic of Korea; Logiq S8, GE Healthcare, Chalfont St. Giles, UK) using linear transducer (>7 MHz) by an experienced examiner blinded to the patients’ clinical data. Following the Mannheim consensus, IMT was measured on the far wall of the common carotid artery in a plaque-free straight 10 mm segment located at least 5 mm proximal to the carotid bifurcation [16,17,29]. IMT was defined as the distance from the intima–lumen to the media–adventitia interface. Two measurements were obtained bilaterally; the mean carotid IMT was calculated as the average of right and left mean values, and the maximum carotid IMT was defined as the greatest value from either side. Carotid plaque was considered present if a focal structure protruded into the lumen by ≥0.5 mm, was ≥50% thicker than the surrounding IMT, or had an absolute thickness >1.5 mm. When plaque was present, the largest plaque was measured with electronic calipers; plaque size-based scoring (≥1.5, ≥2.5, ≥3.5 mm = 1/2/3 points) was recorded.

The PWV was obtained by recording the pulse waves at the carotid and femoral arteries with applanation tonometry connected to an electrocardiogram (ECG) gating system [18]. The path length was determined as the distance between the carotid and femoral measurement sites, adjusted by subtracting the distance from the carotid site to the suprasternal notch from that between the suprasternal notch and the femoral site. The transit time of the pulse wave was calculated as the difference between the R-wave of the ECG and the foot of each pulse wave, and the PWV was computed by dividing the path length by the transit time (m/s). The mean value of two consecutive measurements was used for analysis, and if the difference exceeded 0.5 m/s, an additional measurement was performed, and the median value was adopted.

### 2.4. Measurement of Serum Cholesterol Efflux Capacity

THP-1 cells were cultured in RPMI 1640 medium supplemented with 10% fetal bovine serum (FBS) and harvested to preserve membrane integrity and cholesterol acceptor binding sites. Cellular cholesterol was labeled by incubating the cells with 100 μL of labeling medium for 1 h at 37 °C in the dark. After incubation, the labeling medium was aspirated, and the cells were equilibrated overnight (12–16 h) in 100 μL equilibration medium.

Serum was apoB-depleted by mixing with the kit’s serum treatment reagent at a 2:5 (*v*/*v*) ratio, incubating for 20 min on ice, and centrifuging at 9000× *g* for 10 min at 4 °C. The resulting supernatant was used at a final concentration of 1–4% of the total well volume. Cells were then incubated with each serum for 4 h at 37 °C, 5% CO_2_, protected from light. Following incubation, supernatants (containing effluxed cholesterol) were transferred to a new white 96-well plate. The remaining adherent cells were lysed, and fluorescence intensity of both supernatants and cell lysates was measured using a microplate reader at Ex/Em = 485/523 nm in endpoint mode. Cholesterol efflux was calculated as the fluorescence intensity of the cell culture supernatant divided by the sum of the fluorescence intensity of the supernatant and the intracellular lipid content (ab196985, Abcam, Cambridge, UK).

### 2.5. Statistics Analysis

Variables following a normal distribution, such as age and systolic BP, were described as means and standard deviations and analyzed using the *t*-test. Variables that did not follow a normal distribution, such as the carotid IMT and plaque score, were described as medians and interquartile ranges (Q1, Q3) and analyzed using non-parametric methods, specifically the Mann–Whitney U test. The Spearman correlations are used for two numerical or ordinal variables, and the point-biserial correlations are used when one variable is numeric and the other is dichotomous in correlation analysis between carotid Doppler results, clinical features, and CV risk prediction scores. All statistical analyses were performed using R software, version 4.3.3. Statistically, 0.05 was considered significant.

## 3. Results

### 3.1. Basic Characteristics of Patients with SLE

A total of 104 women (67 female patients with SLE and 37 female HCs) participated in the study, with a median age of 47 years (44–53 and 44–50 years, respectively) (Table 1). The systolic BP and proportion of those taking hypertension medication were higher in SLE groups (123.39 ± 12.75 mmHg and 34.33%) than in the control group (116.38 ± 10.2 mmHg and 2.7%, *p* = 0.005 and <0.001). While total cholesterol levels were lower (176.7 ± 36.51 vs. 190.62 ± 28.36 mg/dL, *p* = 0.047), HDL- and LDL-cholesterol levels did not differ between the SLE and HC group. In the SLE group, 38.8% (26/67), 31.3% (21/67), and 52.2% (35/67) had mucocutaneous, renal, and musculoskeletal manifestations, respectively. The mean disease duration was 105.5 ± 75.2 months, the mean SLEDAI was 4.19 ± 4.43, and 88.1% (59/67) and 47.8% (32/67) were taking hydroxychloroquine and immunosuppressive agents, respectively.

### 3.2. Cardiovascular Risk Scores in Patients with SLE

The estimated 10-year CV risk based on the FRS was 2.98% [2.21–4.77%] in patients with SLE and 2.55% [2.04–3.26%] in HCs (*p* = 0.098). According to the 2013 ACC/AHA risk score, the corresponding values were 0.76% [0.48–1.7%] and 0.65% [0.44–0.96%], respectively (*p* = 0.541). Based on the KRPM, CV risk was significantly higher in patients with SLE (2.23% [1.45–3.6%]) compared to HCs (1.66% [1.3–2.13%], *p* = 0.049) (Table 2).

### 3.3. Carotid Subclinical Atherosclerosis in Patients with SLE

Carotid plaques were identified in 40 patients with SLE (59.7%) and 17 HCs (45.95%, *p* = 0.253), with no significant difference in prevalence between the groups (*p* = 0.253). However, carotid plaque scores were significantly higher in patients with SLE (1.06 ± 2.02) compared with HCs (0.35 ± 1.01, *p* = 0.025). There was no significant difference in mean carotid IMT, maximal IMT, and mean PWV between the two groups.

### 3.4. Comparisons of Cardiovascular Risk Scores Between Patients with SLE with and Without Carotid Plaques

**Figure 1:**
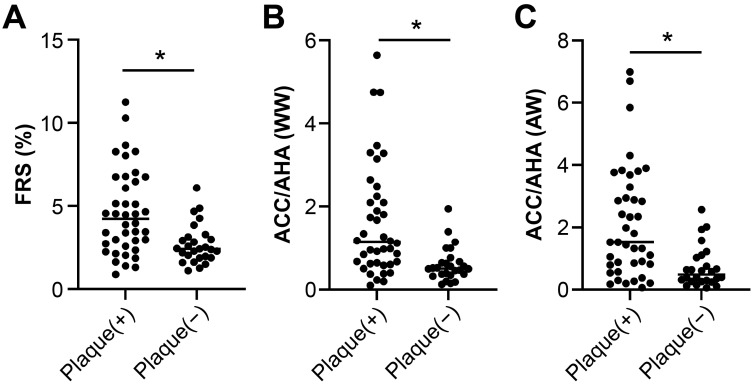
Cardiovascular risk scores in SLE patients according to the presence of carotid plaque. (**A**) Framingham risk score (FRS), (**B**) 2013 ACC/AHA risk score, and (**C**) Korean Risk Prediction Model (KRPM) were all significantly higher in patients with carotid plaque compared to those without (* *p* ≤ 0.001).

The mean FRS score was significantly higher in SLE patients with carotid plaques compared to those without (4.21 [2.7–6.54] vs. 2.41 [1.92–3.06], *p* < 0.001; Figure 1). Similarly, the 2013 ACC/AHA risk score was elevated in SLE patients with carotid plaques compared to those without (1.14 [0.64–2.13] vs. 0.52 [0.28–1.06], *p* < 0.001). The KRPM score also differed significantly between the two groups (2.79 [2.05–4.58] vs. 1.64 [1.31–2.02], *p* = 0.001).

In addition, SLE patient with carotid plaque were older (51 [46–56] years) compared to those without (44 [42–48] years, *p* = 0.002) and had higher BMI (22.49 ± 3.45 kg/m^2^) compared to those without (20.66 ± 2.81 kg/m^2^, *p* = 0.029) (Supplement table). Other clinical features, including manifestations, disease activity, and medication patterns, did not differ between SLE patients with carotid plaque and those without.

### 3.5. Correlations of CV Risk with Subclinical Atherosclerosis in Patients with SLE

The FRS correlated with carotid IMT (r = 0.388, *p* = 0.001), carotid plaque score (r = 0.37, *p* = 0.002), carotid plaque length (r = 0.444, *p* < 0.001), and PWV (r = 0.408, *p* < 0.001) (Table 3). The 2013 ACC/AHA risk score showed similar correlation with carotid IMT (r = 0.397, *p* < 0.001), carotid plaque score (r = 0.388, *p* = 0.001), carotid plaque length (r = 0.456, *p* < 0.001), and PWV (r = 0.429, *p* < 0.001). The KRPM score also correlated with carotid IMT (r = 0.382, *p* = 0.001), carotid plaque score (r = 0.369, *p* = 0.002), carotid plaque length (r = 0.437, *p* < 0.001), and PWV (r = 0.451, *p* < 0.001). Clinical manifestations such as lupus nephritis were not correlated with CV risk scores. In contrast, the use of hydroxychloroquine showed a negative correlation with all risk scores (FRS: r = −0.285, *p* = 0.019, ACC/AHA CV risk: r = −0.328, *p* = 0.007, KRPM: r = −0.263, *p* = 0.031).

### 3.6. Serum Cholesterol Efflux Efficacy Assay in Patients with SLE

**Figure 2:**
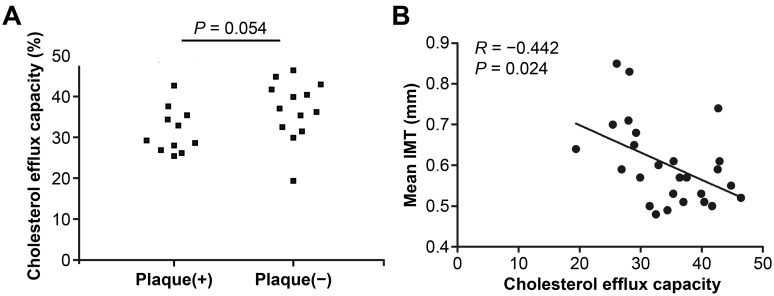
Serum cholesterol efflux capacity (CEC) according to carotid plaque and carotid intima thickness in patients with systemic lupus erythematosus. (**A**) Serum CEC was lower in SLE patients with carotid plaque compared to those without. (**B**) Serum CEC levels were inversely correlated with carotid IMT.

The serum cholesterol efflux capacity (CEC) was 32.2 ± 6.0% in patients with SLE with carotid plaque and 36.8 ± 7.3% in those without (*p* = 0.054), and it was correlated negatively with carotid IMT (r = −0.442, *p* = 0.024) among patients with SLE (Figure 2).

## 4. Discussion

In this study, female patients with SLE had significantly higher CV risk compared to HCs, but only when assessed using the Korean Risk Prediction Model (KRPM). In addition, patients with carotid plaques showed higher CV risk scores across all models, including the FRS, ACC/AHA, and KRPM. Serum cholesterol efflux capacity (CEC) was lower in patients with carotid plaque and showed a significant negative correlation with carotid IMT.

When comparing CV risk scores, only the KRPM identified a significant difference between patients with SLE and HCs. This may be explained by the fact that KRPM was developed using Korean epidemiological data, which better reflects population-specific risk factor distributions. These findings suggest that KRPM may be a more suitable tool for assessing CV risk in Korean patients with SLE. In contrast, other studies have shown that modified FRS and QRISKs (cardiovascular disease risk prediction algorithm) are useful for predicting CV risk in SLE, and QRISK3 has been highlighted as having strong potential for estimating the 10-year risk of CVD [30,31]. For example, patients with SLE and high QRISK3 scores had higher PWV and mean cIMT values than those with low scores [32]. Bolla et al. also reported that hypertension, obesity, and hyperlipidemia are common in SLE, and that the frequency of risk factors varies across countries depending on income levels [2]. Recently, Choi et al. developed SLECRISK, a new SLE-specific model, which predicts the 10-year risk of major adverse cardiovascular events in young females with severe SLE [33]. This model includes SLE-specific parameters, such as SLEDAI, disease duration, autoantibodies, and complement levels, and has shown better predictive value than the FRS or ACC/AHA. However, it requires detailed clinical information and cannot be applied to compare patients with SLE to healthy controls. In contrast, KRPM relies on routine clinical parameters and is therefore more accessible for general clinical use.

Studies that assess subclinical atherosclerosis within vessels can provide useful information regarding CV risk in SLE, and several studies have shown increased carotid IMT and a higher prevalence of carotid plaque among patients with SLE than in the general population [10,34,35]. Moreover, carotid IMT and plaques can predict future CV events in patients with SLE without previous CV events [36,37,38]. In a study of patients with SLE, 36% patients had carotid or femoral plaques, and increased echogenicity was associated with taking prednisolone and persistent disease activity [39]. In this study, patients with SLE and carotid plaques had higher risk scores for CVD, and carotid IMT and carotid plaque length were correlated to the CV risk prediction scores of the FRS, 2013 ACC/AHA, and KRPM. The prediction models showed a correlation with subclinical atherosclerosis, which was assessed using carotid Doppler ultrasonography. However, the degree of association between the scores of the risk models and carotid IMT and the presence of plaque was not sufficient (OR 0.3–0.5), suggesting that the prediction models, including additional tests such as carotid ultrasonography, may be more accurate than the tools we currently use.

In addition, taking hydroxychloroquine was associated with a lower risk for CVD according to the FRS, 2013 ACC/AHA, and the KRPM. Hydroxychloroquine is known to reduce blood pressure variability, but its association with structural parameters such as carotid IMT, carotid plaque, and coronary artery calcification has not been determined [40]. In our analysis, the use of hydroxychloroquine was negatively correlated with all risk scores for CVD but not with the carotid Doppler results or PWV.

Atherosclerosis progression is influenced by cholesterol metabolism, and chronic inflammatory conditions impair the anti-thrombotic and antioxidant functions of HDL [19,20]. HDL dysfunction can be evaluated by the cholesterol efflux capacity in serum, which is reduced in SLE compared with healthy controls and RA patients [23,25]. In this study, serum cholesterol efflux capacity was lower in patients with carotid plaques, although not statistically significant, and negatively correlated with carotid IMT. The observed inverse correlation between carotid IMT and cholesterol efflux capacity can be explained by the role of cholesterol efflux capacity as a functional measure of HDL. A reduced cholesterol efflux capacity indicates impaired HDL-mediated removal of intracellular cholesterol, leading to increased cholesterol accumulation within vascular cells. This accumulation promotes the transformation of endothelial and macrophage cells into foam cells, thereby accelerating atherosclerotic plaque formation and vascular wall thickening, which is reflected as increased carotid IMT. To our knowledge, this study is the first data to confirm the correlation between actual atherosclerosis indicators and impaired HDL function.

This study has several limitations. The number of SLE patients was small, and only female participants were included. In addition, CEC could not be assessed in all subjects, which may reduce the reliability of the findings. Furthermore, this study did not evaluate actual clinical outcomes such as cardiovascular events. Nevertheless, by examining both CVD risk models and subclinical atherosclerosis, we provide useful insight into their clinical applicability. Our findings confirm the utility of KRPM for Korean patients with SLE and highlight the need for tailored CVD prediction tools. Finally, the observed association between impaired HDL function and carotid IMT underscores the role of dysfunctional HDL in the pathogenesis of atherosclerosis in SLE.

## 5. Conclusions

This study demonstrated that Korean female patients with SLE have an increased cardiovascular risk compared to healthy controls, particularly when assessed using the Korean Risk Prediction Model (KRPM). The presence of carotid plaques was associated with higher risk scores across multiple prediction models, and cholesterol efflux capacity showed a negative correlation with carotid IMT, suggesting that impaired HDL function may contribute to the development of subclinical atherosclerosis in SLE. These findings underscore the value of incorporating both population-specific risk prediction models and vascular imaging into CVD risk assessment in SLE. Larger, longitudinal studies are warranted to validate these results, clarify causal relationships, and guide the development of SLE-tailored cardiovascular risk prediction tools for improved patient care.

## Figures and Tables

**Table 1:** Baseline characteristics of objectives.

Characteristics	SLE (*n* = 67)	HC (*n* = 37)	*p*
Age, years	47 (44, 53)	47 (44, 50)	0.596
Systolic blood pressure, mmHg	123.39 ± 12.75	116.38 ± 10.2	0.005
Diastolic blood pressure, mmHg	72.34 ± 8.89	70.14 ± 9.6	0.169
Hypertension medication, *n* (%)	23 (34.33)	1 (2.7)	<0.001
Weight, kg	53 (48.25, 60.83)	58.3 (53.4, 64.5)	0.01
Height, cm	159 (155, 161.75)	159.7 (157, 162)	0.197
Body mass index, kg/m^2^	21.75 ± 3.31	22.97 ± 2.64	0.057
Total cholesterol, mg/dL	176.7 ± 36.51	190.62 ± 28.36	0.047
HDL cholesterol, mg/dL	61.27 ± 14.12	61.24 ± 14.84	0.993
LDL cholesterol, mg/dL	105.4 ± 33.2	106.6 ± 24.5	0.533
Mean carotid IMT, mm	0.6 (0.55, 0.65)	0.61 (0.56, 0.66)	0.881
Maximal carotid IMT, mm	0.67 (0.61, 0.73)	0.73 (0.67, 0.79)	0.21
Carotid plaque, *n* (%)	40 (59.7)	17 (45.95)	0.253
Carotid plaque score	1.06 (2.02)	0.35 (1.01)	0.025
Carotid plaque length, mm	1.2 (0, 3.9)	0 (0, 1.2)	0.054
Mean PWV, m/s	6.05 ± 0.96	5.87 ± 0.72	0.321
Disease duration, months	105.5 ± 75.2		
Mucocutaneous involvement, *n* (%)	26 (38.8)		
Renal involvement, *n* (%)	21 (31.3)		
Musculoskeletal involvement, *n* (%)	35 (52.2)		
Hematologic involvement, *n* (%)	23 (34.3)		
Complement deficiency, *n* (%)	34 (50.7)		
SLEDAI	4.19 ± 4.43		
Cumulative dose of glucocorticoids, g	1.6 ± 3.2		
HCQ, *n* (%)	59 (88.1)		
Immunosuppressive therapy, *n* (%)	32 (47.8)		
NSAID, *n* (%)	28 (41.8)		
Aspirin, *n* (%)	5 (7.5)		

SLE, systemic lupus erythematosus; HC, healthy control; HDL, high-density lipoprotein; LDL, low-density lipoprotein; IMT, intima-media thickness; PWV, pulse wave velocity; ACC/AHA, the American College of Cardiology/American Heart Association; KRPM, the Korean Risk Prediction Model; SLEDAI, the SLE disease activity index; NSAID, nonsteroidal anti-inflammatory drugs.

**Table 2:** Cardiovascular risk scores in patients with systemic lupus erythematosus and healthy control.

Prediction Models	SLE	HC	*p*
Framingham risk score, %	2.98 (2.21, 4.77)	2.55 (2.04, 3.26)	0.098
ACC/AHA CV risk, %	0.76 (0.48, 1.7)	0.65 (0.44, 0.96)	0.541
KRPM, %	2.23 (1.45, 3.6)	1.66 (1.3, 2.13)	0.049

ACC/AHA, the American College of Cardiology/American Heart Association; KRPM, the Korean Risk Prediction Model.

**Table 3:** Correlations of CVD risk and carotid Doppler and PWV in patients with systemic lupus erythematosus.

	Framingham		2013 ACC/AHA		KRPM	
	r	*p*-Value	r	*p*-Value	r	*p*-Value
Carotid IMT	0.388	0.001	0.397	<0.001	0.382	0.001
Carotid plaque score	0.37	0.002	0.388	0.001	0.369	0.002
Carotid plaque length	0.444	<0.001	0.456	<0.001	0.437	<0.001
PWV	0.408	<0.001	0.429	<0.001	0.451	<0.001
Mucocutaneous involvement	−0.244	0.046	−0.174	0.159	−0.19	0.123
Renal involvement	0.167	0.176	0.119	0.336	0.124	0.317
Musculoskeletal involvement	−0.124	0.316	−0.056	0.655	−0.069	0.581
Hematologic involvement	−0.203	0.099	−0.213	0.084	−0.175	0.156
Complement deficiency	−0.19	0.124	−0.182	0.414	−0.165	0.182
SLEDAI	−0.057	0.649	−0.062	0.62	−0.096	0.439
Total dose of GCs	0.158	0.201	0.147	0.236	0.138	0.267
Hydroxychloroquine	−0.285	0.019	−0.328	0.007	−0.263	0.031
Immunosuppressive therapy	0.006	0.963	−0.015	0.903	−0.039	0.756
NSAID	0.041	0.743	0.091	0.464	0.09	0.468
Aspirin	−0.051	0.681	−0.005	0.968	0.011	0.93

ACC/AHA, the American College of Cardiology/American Heart Association; WW, white women; AW, African white; KRPM, the Korean Risk Prediction Model; IMT, intima-media thickness; PWV, pulse wave velocity; SLEDAI, the SLE disease activity index; GCs, glucocorticoids; NSAID, nonsteroidal anti-inflammatory drug.

## Data Availability

The raw data supporting the conclusions of this article will be made available by the authors on request.
